# The antiviral BDGR-49 provides protection from lethal, neurotropic Venezuelan equine encephalitis virus intranasal infection in mice

**DOI:** 10.1128/jvi.01679-24

**Published:** 2025-02-12

**Authors:** Evan P. Williams, Yi Xue, Peter Vogel, Dong Yang, Alejandro Ponce-Flores, Xiaoyu Li, Tyler J. Ogorek, Manisha Saini, Jorge Iulek, Francesc Xavier Ruiz, Eddy Arnold, Jennifer E. Golden, Bernd Meibohm, Colleen B. Jonsson

**Affiliations:** 1 Department of Microbiology, Immunology and Biochemistry, College of Medicine, The University of Tennessee Health Science Center 274062 , Memphis, Tennessee, USA; 2 Animal Resources Center and Veterinary Pathology Core, St. Jude Children's Research Hospital 5417 , Memphis, Tennessee, USA; 3 Regional Biocontainment Laboratory, The University of Tennessee Health Science Center 12326 , Memphis, Tennessee, USA; 4 Division of Pharmaceutical Sciences, School of Pharmacy, University of Wisconsin-Madison 15533 , Madison, Wisconsin, USA; 5 Department of Chemistry, University of Wisconsin-Madison 201643 , Madison, Wisconsin, USA; 6 Center for Advanced Biotechnology and Medicine, Rutgers The State University of New Jersey 242612 , Piscataway, New Jersey, USA; 7 Department of Chemistry and Chemical Biology, Rutgers The State University of New Jersey 242612 , Piscataway, New Jersey, USA; 8 Department of Chemistry, State University of Ponta Grossa 67883 , Ponta Grossa, Brazil; 9 Department of Pharmaceutical Sciences, College of Pharmacy, The University of Tennessee Health Science Center 427811 , Memphis, Tennessee, USA; 10 Institute for the Study of Host-Pathogen Systems, The University of Tennessee Health Science Center 12326 , Memphis, Tennessee, USA; University of Kentucky College of Medicine, Lexington, Kentucky, USA

**Keywords:** Venezuelan encephalitis virus, eastern equine encephalitis virus, western equine encephalitis virus, antiviral agents, host response, cell death, pathology, immune response, mouse, BDGR-49

## Abstract

Venezuelan, western, and eastern equine encephalitis virus (VEEV, WEEV, and EEEV) cause a febrile illness that may result in fatal neurological disease in humans and equines. Human infections are typically from mosquito bites, although cases from respiratory exposure in laboratory accidents have been documented. In addition to natural mosquito-borne infection, the potential biothreat inherent in the ability to disseminate these viruses via the respiratory route has driven the development of antiviral drugs for this route of exposure. To address this gap, we tested the prophylactic administration of a novel brain-penetrant, antiviral, BDGR-49, against a lethal intranasal challenge of VEEV, WEEV, or EEEV in BALB/c mouse model. BDGR-49 conferred 100% protection with 6 mg kg^−1^ twice per day for 6 days for VEEV, but not EEEV or WEEV. By 8 days post-infection (dpi), infectious virus, viral RNA, and viral antigen in the brain of BDGR-49-treated mice were significantly reduced. Brains of VEEV TrD-infected, BDGR-49-treated mice showed a significant reduction in the expression of genes associated with inflammation (*IFNB1*, *TNF*, *IL6*, and *CCL5*) and cell death (*CASP4*, *GSDMD*, *PYCARD*, and *ZBP1*). At dpi 14, histopathology showed that neuronal lesions and inflammatory cell infiltrates were essentially absent, and viral antigen was not detected in the brains of VEEV TrD-infected, BDGR-49-treated mice. In summary, although BDGR-49 treatment showed significant promise for the treatment of mice exposed intranasally to VEEV, the more rapid and efficient entry of EEEV and WEEV by this route into the central nervous system will require additional optimization of the dosing regimen.

**IMPORTANCE:**

Prophylactic and therapeutic treatment of viruses that cause encephalitis requires fast-acting drugs that rapidly penetrate the blood-brain barrier. Currently, clinicians have only a limited set of antivirals for the treatment of neurotropic infections such as herpesviruses or HIV-1, and none for alphaviruses, and treatment outcomes remain poor. New medical countermeasures will address the gap in treatment of viral encephalitis such as those caused by the neurotropic alphaviruses and others.

## INTRODUCTION

Mosquito-borne infections of Venezuelan, western, or eastern equine encephalitis virus (VEEV, EEEV, and WEEV) in humans and equines arise from epidemic and epizootic cycles in America (1, 2). In addition to mosquito-borne infections, human cases have been documented from respiratory exposure in laboratory accidents and in animal studies, with potentially higher mortality than with natural infections (3–5). Common clinical symptoms and laboratory findings from either route of exposure include biphasic fever, lethargy, viremia, and lymphopenia (5–8). In children and older adults, severe neurological disease can occur with convulsions, seizures, stupor, coma, and the possibility of death (5, 9). Pathogenesis results from lymphocyte destruction, endothelial cell injury, and viral encephalitis (6, 8, 10, 11). Based on the historical biowarfare threat posed by VEEV, the ability to disseminate these viruses via the respiratory route, their ability to grow to high titer in cell culture, and the lack of therapeutics for treatment, VEEV and EEEV are designated as select agents by the Center for Disease Control and Prevention and Category B priority pathogens by the National Institutes of Health (12, 13).

In respiratory-acquired infections in mice and humans, VEEV, EEEV, and WEEV enter the central nervous system (CNS) via the bipolar olfactory neurons, whose axons form the olfactory nerves, which transmit the virus directly to the olfactory bulbs via axonal transport, with the virus infection then spreading rapidly to other regions of the brain (14, 15). In mouse models of encephalitic alphaviruses, tissue damage due to infection and destruction of neurons is notable (11, 16) and is accompanied by an influx of natural killer (NK) cells, T cells, neutrophils, and monocytes into the CNS (17) and an increase in expression of pro-inflammatory cytokines and chemokines (18), which may contribute to neurologic disease (19). Several lines of evidence have suggested that the CD4^+^ T cells that infiltrate the brain are protective (12, 20), whereas NK cells increase pathology (21).

Treatment of viral encephalitis requires antivirals that cross the blood-brain barrier, reach the sites within the brain where viruses reside, prevent viral replication, and slow the disease course. Therefore, in addition to the evaluation of the biodistribution and intracellular pharmacokinetics of a brain-penetrating antiviral, the assessment of antivirals during lead optimization and development demands greater mechanistic insight into the underlying spatial-temporal dynamics of virus spread, virus-host interactions, and disease progression. Herein, we focused on the CNS component of disease using a model of intranasal infection of BALB/c mice with VEEV strain Trinidad Donkey (TrD) with and without BDGR-49 treatment (22). BDGR-49 is a potent, quinazolinone-based antiviral compound with demonstrated prophylactic and therapeutic protection from subcutaneous infection of BALB/c mice with VEEV TrD (22). We chose to use the intranasal rather than the subcutaneous route of infection to achieve higher levels of virus in the brain of the mice and thereby focus our efforts on treatment of the CNS component of disease. Prior studies to define a mechanism of action showed that BDGR-49 effectively inhibits plus and minus strand synthesis of VEEV (22–24). Additionally, *in vitro* antiviral resistance assays that used a 2-fold escalation of BDGR-49 in VEEV TC-83-infected Vero 76 cells revealed resistance mutations in the N terminal regions of nsP2 (Y102C, D116N, and E118V) and nsP4 (Y87F, Q210R, and Q201K) (22). Together with the inhibition of viral RNA synthesis, the location of these amino acid changes suggests that BDGR-49 may antagonize the interaction of these two proteins within the replication machinery as its mechanism of action. Prediction of the structure of the VEEV nsP2/nsP4 complex using Alphafold2, followed by molecular docking, supports the notion that BDGR-49 binds at the interface between both proteins, which might impair the polymerase activity of nsP4 and hence viral RNA synthesis. Herein, we evaluated the efficacy of BDGR-49 (22) in lethal, intranasal infection models of BALB/c mice with VEEV, WEEV, or EEEV. Moreover, we probed the host transcriptome, viral RNA levels, and viral load and pathology in the brain over time in the presence and absence of BDGR-49 in VEEV-infected, or mock-exposed, BALB/c mice. Finally, we evaluated potential resistance mutations of VEEV TrD to BDGR-49 in mouse brains using a replicon assay.

## MATERIALS AND METHODS

### Cells and viruses

VEEV TrD was purchased from BEI (NR-332), and WEEV Fleming and EEEV FL93-939 were obtained from the World Reference Center for Emerging Viruses and Arboviruses (WRCEVA, UTMB-Galveston, TX). VEEV TC-83 was obtained from Dr. Connie Schmaljohn (United States Army Medical Research Institute of Infectious Diseases, Maryland). Virus seed stocks were amplified in either Vero 76 (ATCC CRL-1587) or BHK-21 (ATCC CCL-10) cells in Dulbecco’s modified essential medium or minimum essential medium with Earle’s modification with 10% fetal bovine serum, 1% penicillin-streptomycin, and 1% L-glutamine and clarified twice by low-speed centrifugation. All cell culture reagents were purchased from ThermoFisher Scientific unless otherwise specified. The seed stock of VEEV TrD was sequenced as a reference genome for bioinformatic analyses (25).

### Plaque assay

Twelve-well tissue culture plates were seeded with 2.5 × 10^5^ Vero 76 cells, and a series of 10-fold dilutions of samples were made to measure infectious viral titers as described previously (26).

### BDGR-49 synthesis

**Fig 1:**
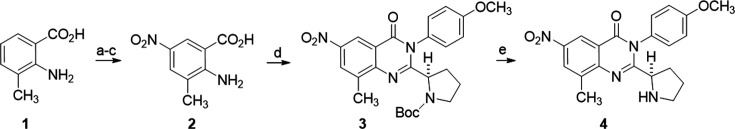
Second generation synthesis of BDGR-49 that spares enantiopurity. Reagents and conditions: (a) Ac_2_O, reflux, 2 h, 68%; (b) H_2_SO_4_, HNO_3_, 76% (c) conc. HCI, reflux, 3 h, 94%; (d) *N*-Boc D-proline, MsCI, NMI, CuCl_2_, DCE, 0 °C to rt, 3 h; then anthranililic acid 2, rt, 24 h, then TMSCI, NEt_3_, reflux, 24 h, 60%; (e) TFA, DCM, 0 °C to rt, 2 h, 77%.

**Fig 2:**
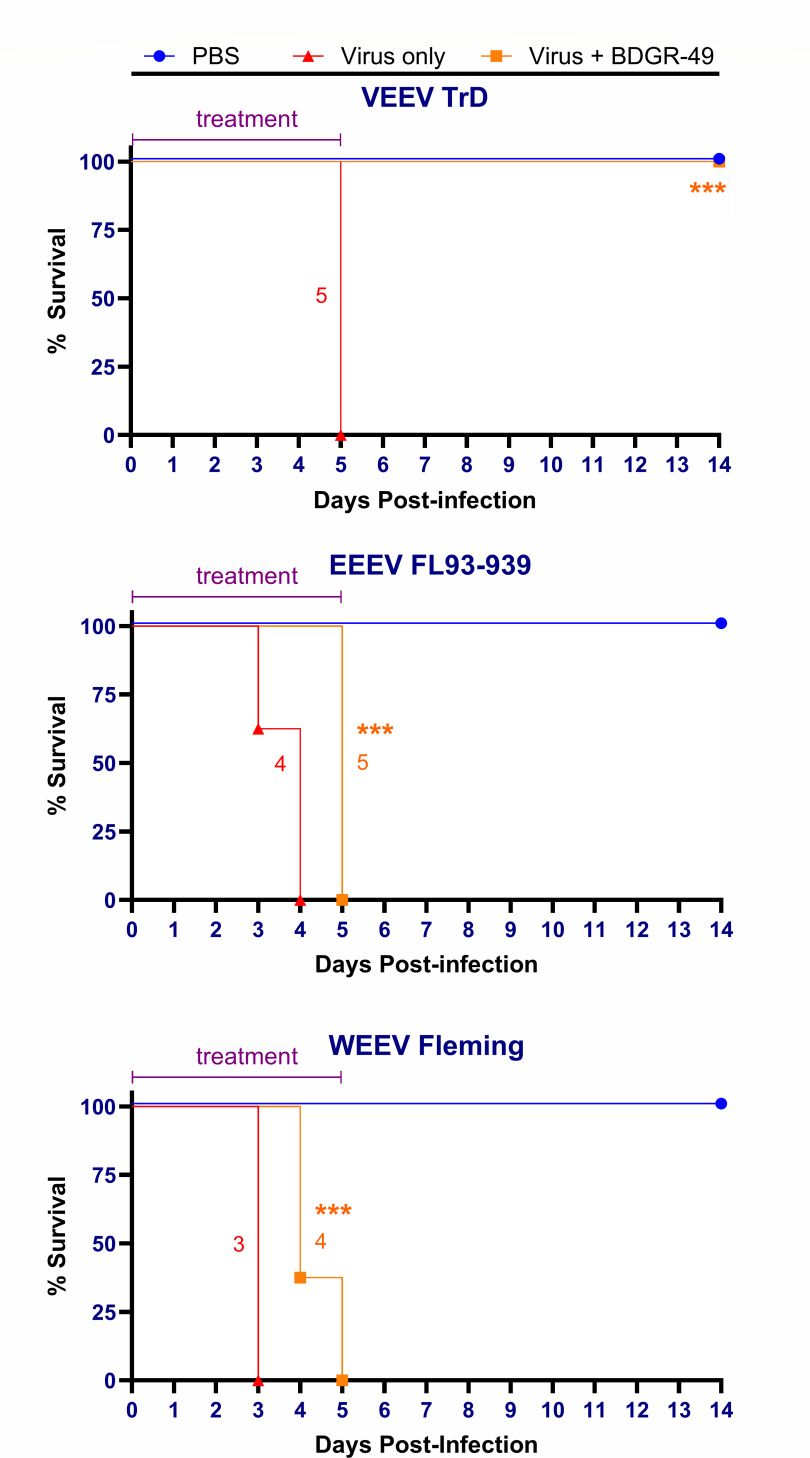
Survival of BALB/c mice intranasally infected with VEEV TrD, EEEV FL-93–939, WEEV-Fleming, or Mock and treated prophylactically with BDGR-49 or sham. Survival curves are presented for VEEV, EEEV, and WEEV in mice (*n* = 8 per group) infected intranasally with PBS or virus and treated with sham or BDGR-49. Female BALB/c mice at 6–7 weeks of age were treated by subcutaneous administration of 6 mg kg^−1^ of BDGR-49 at 2 h before intranasal challenge with 10 × LD_50_ of virus. A second dose of 6 mg kg^−1^ BDGR-49 was given 10 h after infection, and dosing was continued twice daily at 12 h intervals for an additional 5 days. The median survival (days) is provided adjacent to those lines in which mice succumbed to infection. The survival curves for each group were compared using the log-rank (Mantel-Cox) test and those survival curves that were significantly different are shown with asterisk marks (***, *P* value 0.001).

**Fig 3:**
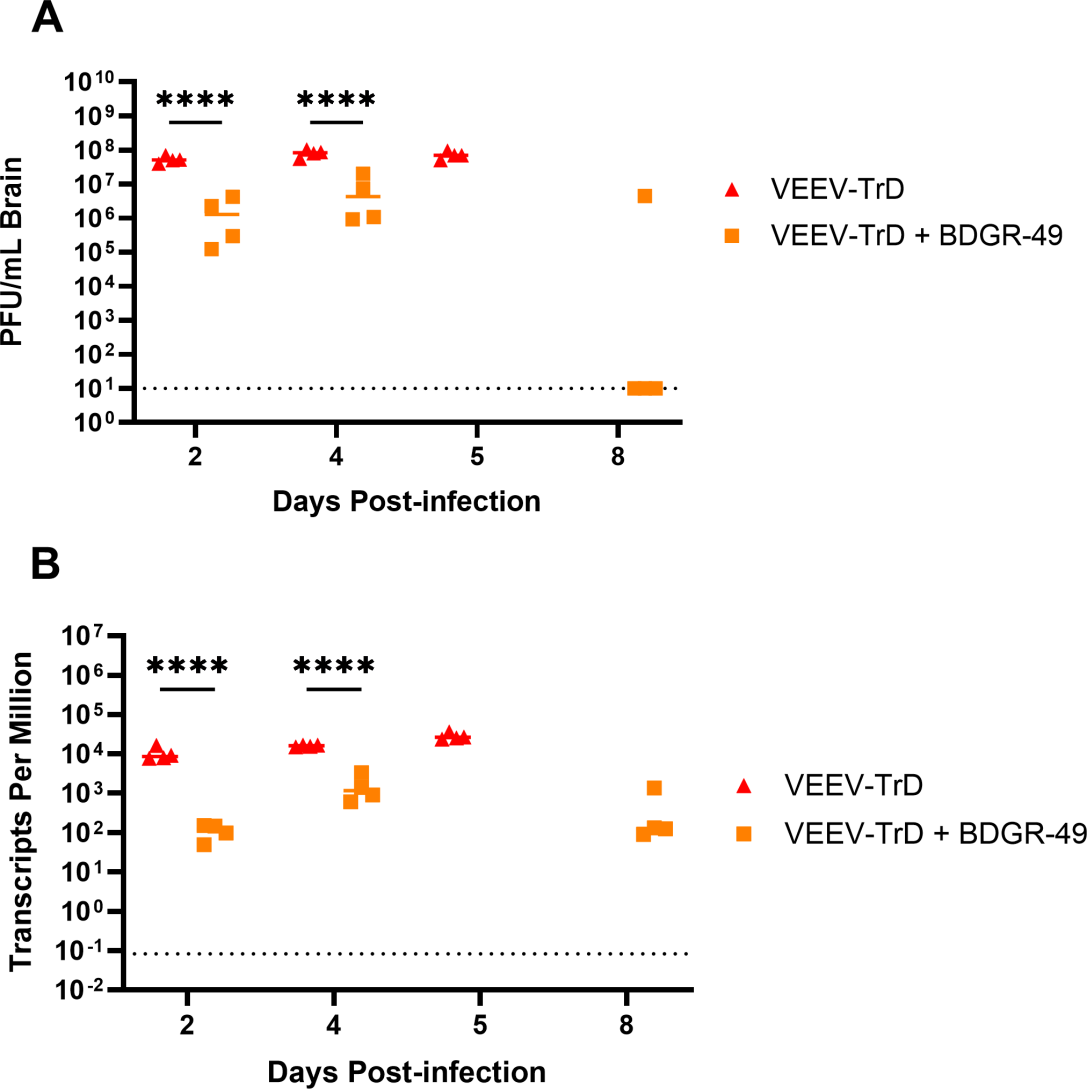
Quantification of virus present in the brains of mice intranasally infected with VEEV-TrD treated with sham or BDGR-49. On 2, 4, 5 (sham-treated only), and 8 (BDGR-treated only) dpi, four mice from each group were euthanized, and brains were harvested. Brains were homogenized in PBS, and the homogenates were used to quantify (**A**) infectious virus levels using the plaque assay (**B**) and viral genome transcripts per million by RNA-seq. As described in the Material and Methods, total RNA from each brain was isolated, used to prepare libraries for RNA-seq, and sequenced on an Illumina sequencing platform. Sequencing reads were mapped to a concatenated genome reference for mouse and virus and transcripts per million assessed. Statistical significance was determined by using a 2-way ANOVA multiple comparison analysis with Šidák multiple comparison correction.

**Fig 4:**
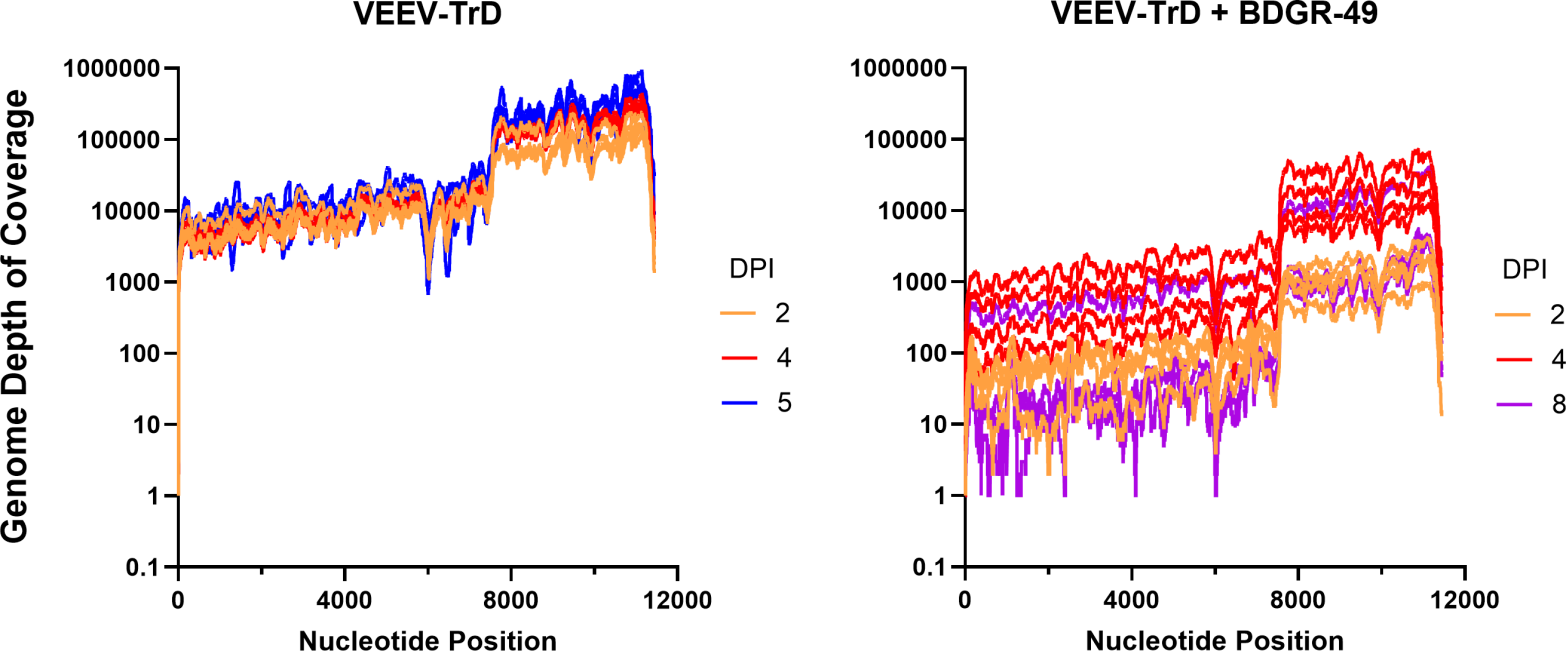
Depth of coverage of VEEV genomes in the brains of mice infected intranasally with VEEV-TrD treated with sham or BDGR-49. On 2, 4, 5 (sham-treated only), and 8 (VEEV TrD + BDGR-treated only) dpi, four mice from each group were euthanized, and brains were harvested and homogenized. As described in the Materials and Methods, total RNA from each brain was isolated and used to prepare libraries for RNA-seq and sequenced on an Illumina sequencing platform. Sequencing reads were mapped to a concatenated genome reference for mouse and virus, and the viral genome depth of coverage was determined. The individual lines represent the depth of nucleotide (Nt) coverage of viral genomes for each mouse brain at 2 (orange lines), 4 (red lines), 5 (blue lines), or 8 dpi (purple lines) in groups that were (**left**) sham-treated (PBS) and infected with VEEV-TrD or (**right**) BDGR-49-treated and infected with VEEV-TrD. The x-axis corresponds to the nucleotide position within the full-length viral genomes. The depth of coverage represents the number of reads that mapped to a concatenated genome with the mouse virus as described in the Materials and Methods.

**Fig 5:**
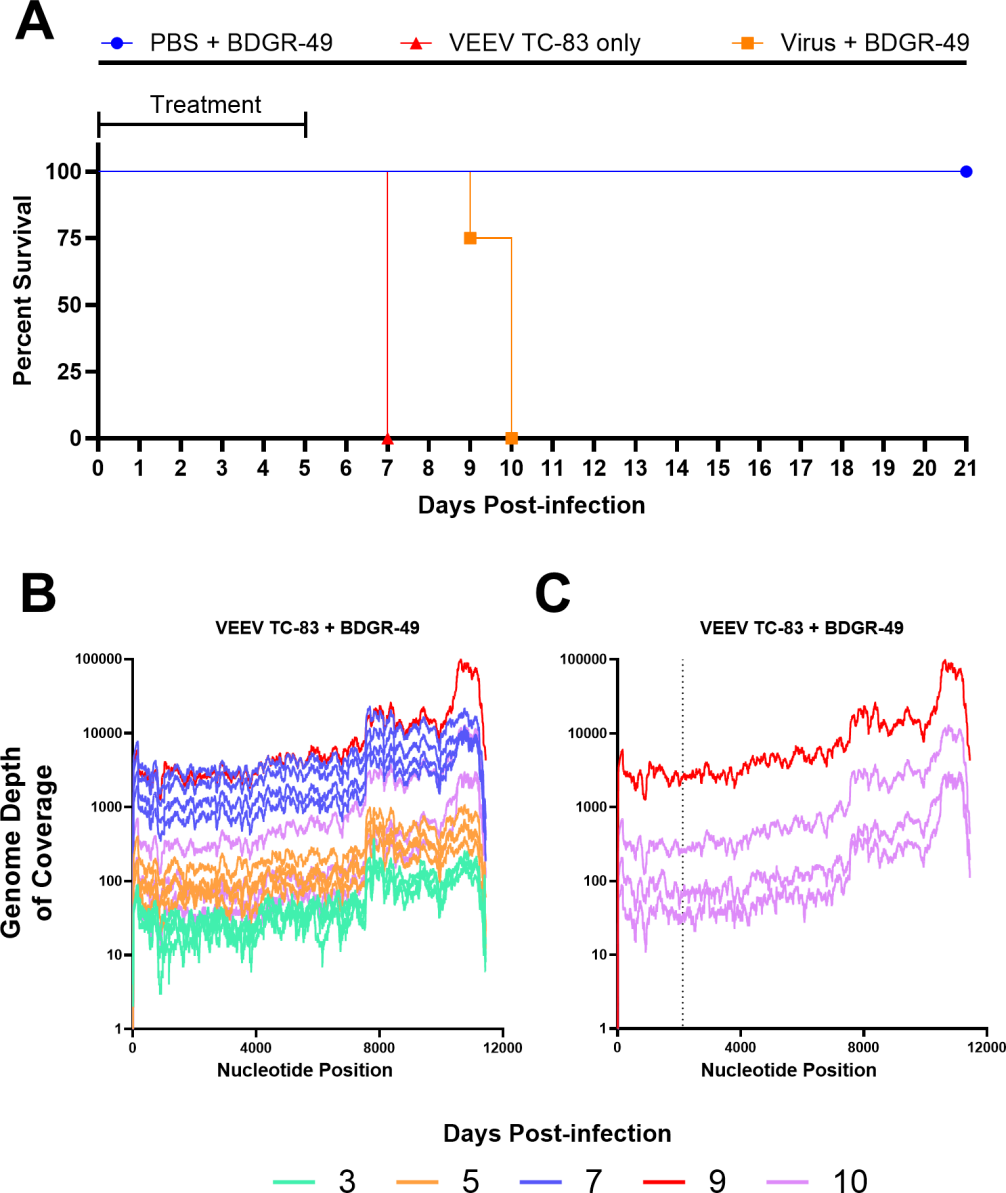
Suboptimal prophylactic treatment of C3H/HeN mice with BDGR-49 or sham and challenged with VEEV TC-8. (**A**) Survival curves are presented for mice (*n* = 8 per group) infected intranasally with PBS or VEEV TC-83 and treated with sham or BDGR-49. Mice at 6–8 weeks of age were treated by subcutaneous administration of 6 mg kg^−1^ of BDGR-49 at 2 h before intranasal challenge with 1 × 10^7^ PFU of virus, which is lethal in C3H/HeN mice (36). A second dose of 6 mg kg^−1^ BDGR-49 was given 10 h after infection, and dosing was continued twice daily at 12 h intervals for an additional 5 days. (**B**) Depth of coverage of VEEV TC-83 genomes from RNA-seq of total RNA from brains of mice intranasally infected with VEEV TC-83 and treated with BDGR-49 as described in the Methods. On 3, 5, 7, and 10 dpi, four mice from each group were euthanized, and the brains were harvested, homogenized, and total RNA was sequenced using RNA-seq. One mouse succumbed to infection on nine dpi and is highlighted in red. (**C**) Depth of coverage of VEEV TC-83 genomes from 9 and 10 dpi are highlighted. The genome from the brain from 9 dpi had a mutation at NSP2:L156Q. Dotted line indicated nucleotide position 2116, where the nucleotide change occurred resulting in the mutation NSP2:L156Q.

**Fig 6:**
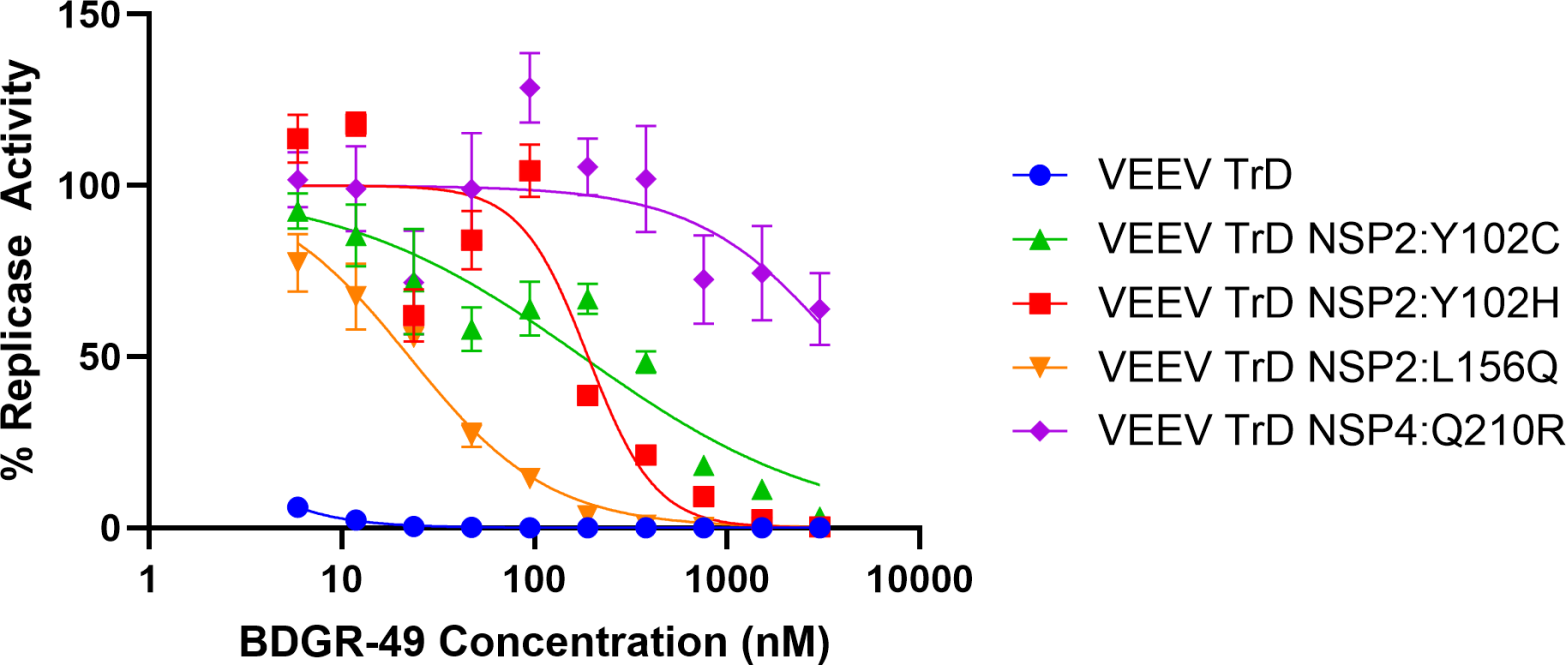
Evaluation of NSP2:Y102H and NSP2:L156Q mutations identified in mouse brains from VEEV TrD-infected mice treated with BDGR-49. Replication activity of VEEV TrD nsP1234 in the absence or presence of BDGR-49 was determined using an *Alphavirus trans-*replicon system as described in Materials and Methods. Briefly, 293T cells were transfected with one plasmid to produce the nsP1234 replicase complex (wildtype or mutations) and one plasmid with the two reporters in the presence of varying concentrations of BDGR-49. The activity of replicase complexes of wildtype or mutations was normalized to the negative control (plasmid with mutations in the nsP4 at the active site) in varying concentrations of BDGR-49. Percentage replicase activity curves were used to calculate IC_50_ values for wildtype or mutated replication complexes. Replication complexes assessed were wildtype (blue, circles), nsP2:Y102C (green triangle), nsP2:Y102H (red squares), nsP2:L156Q (orange triangles), and nsP4:Q210R (purple diamond).

BDGR-49 was synthesized by one of the two protocols, generating the compound with >98% analytical purity by liquid chromatography/mass spectrometry (LCMS) but in different enantiopurities. The primary synthesis used to construct BDGR-49 was previously published (22), furnishing the compound in 82% enantiomeric excess (ee) due to limited racemization in the quinazolinone assembly step. This batch was used for data depicted in Fig. 1 to 3, 6, and 7. To improve the enantiopurity, a new synthetic method (27) was developed, which preserved the enantiopurity of the N-Boc proline material and provided BDGR-49 in >99% ee. The material of higher enantiopurity (>99%) was generated according to Fig. 4 and was used to generate the data shown in Fig. 5 and 6; Table 1; Fig. 8 to 10, Supplement 1. The second-generation synthesis of BDGR-49 involved the acylation of aniline 1, followed by nitration and deacetylation to provide nitroaniline 2 (Fig. 1). Using a methodology (27) designed to preserve enantiopurity, the quinazolinone core was assembled to provide N-Boc protected intermediate 3 in 60% yield. Removal of the protecting group with TFA, followed by neutralization afforded BDGR-49 in 77% yield and with >99% ee as determined by chiral HPLC. Regardless of the protocol employed, the final compound was identical in terms of previously published ^1^H, ^13^C, and ^19^F NMR spectra and high-resolution mass (HRMS) spectroscopy data.

### General animal study information and compliance for all studies

Female, BALB/c mice, or C3H/HeN mice, aged 6–8 weeks old were obtained from Charles River Laboratories and randomized. As we have not noted any difference between male and female mice in treatment outcomes, we have not yet included both in all studies but rather have focused on female mice cohorts. In all studies, mice were infected intranasally at 10^7^ PFU for VEEV TC-83 or 10× the lethal dose at 50% (LD_50_), which was estimated at 7,500 PFU of VEEV TrD, 7,500 PFU EEEV FL93-939, or 5,000 PFU WEEV Fleming, or mock inoculated with Dulbecco’s’ phosphate buffered saline (PBS) using 15 µL per naris. After inoculation, all mice were weighed daily and checked twice daily for clinical signs and morbidity until recovery. For all studies, BDGR-49 was formulated in 25% PEG400/10% Kolliphor RH40/65% water as previously described (22). All mice were humanely sacrificed using isoflurane followed by cervical dislocation.

### Mouse study design for survival studies

BALB/c mice (*n* = 8/group) were intranasally infected with VEEV TrD, EEEV FL93-939, or WEEV Fleming and treated twice per day with either PBS or with 6 mg kg^−1^ of BDGR-49. The control group of BALB/c mice (*n* = 8) was not infected nor treated. Similarly, the C3H/HeN mice (*n* = 8/group) were intranasally infected with VEEV TC-83 and treated twice per day with either PBS or with 6 mg kg^−1^ of BDGR-49. Treatments were administered by subcutaneous injection starting at −2 h prior to infection on day 0 through day 5 for a total of 6 days. Mice were monitored for mortality and clinical signs twice a day until they met the criteria for euthanasia or 14 dpi.

### Mouse study design and sample collection for plaque assay and RNA-seq

BALB/c mice were intranasally infected with VEEV TrD or VEEV TC-83 and treated twice per day with either PBS (*n* = 12) or with 6 mg kg^−1^ of BDGR-49 (*n* = 12). The control group of BALB/c or C3H/HeN mice (*n* = 12) were not infected nor treated. Treatments were administered by subcutaneous injection starting at −2 h prior to infection on day 0 through day 5 for a total of 6 days. Mice were monitored for mortality and clinical signs twice a day. At 2, 4, 5 (mock-treated), and 8 (BDGR-49 treated group only) dpi, four mice per group were euthanized, and their brains were collected for plaque assay and RNA-seq.

### RNA-seq of brains from BALB/c mice with VEEV TrD and BDGR-49 or sham

Total RNA was isolated from each brain using TRIzol Reagent and was further purified using DNase treatment using the TURBO DNA-*free* Kit (Invitrogen). Total RNA was quantified and qualified using the BioAnalyzer 2100 (Agilent), whereafter sequencing libraries were constructed by mRNA enrichment using Illumina TruSeq Stranded mRNA Library Prep Kit (Illumina). Constructed libraries were sequenced on Illumina’s HiSeq platform by Novogene. Sequence read results were processed, trimmed, and filtered, using default parameters with CLC Genomics Workbench (Qiagen, v23). Reads were mapped to default parameters against concatenated reference genomes consisting of *Mus musculus* (Ensembl, GRCm38) and our sequenced lab strain of VEEV TrD. Gene count results were used to assess differential gene expression using DESeq2, and mock-inoculated mice-defined baseline gene expression. RNA-seq unprocessed and processed data are available on NIH Gene Expression Omnibus under the series GSE255795 and GSE277300.

### RNA-seq of brains from C3H/HeN mice with VEEV TC-83 and BDGR-49 or sham

Total RNA was isolated from each brain using the MagMax mirVana Total RNA Isolation Kit and the KingFisher Flex System (Thermo Fisher Scientific). Library preparation and sequencing were performed by Azenta Life Sciences. The quantity and quality of total RNA were assessed by Qubit and TapeStation. Sequencing libraries were constructed by mRNA enrichment using NEBNext Ultra II RNA Kit (New England Biolabs). Prepared libraries were sequenced on Illumina’s HiSeq 4000 platform (2 × 150, 40 million pair-end reads per sample). Sequence read results were processed, trimmed, and filtered, using default parameters with CLC Genomics Workbench (Qiagen, v23). Reads were mapped to default parameters against concatenated reference genomes consisting of *Mus musculus* (Ensembl, GRCm38) and our sequenced lab strain of VEEV TC-83. RNA-seq unprocessed and processed data are available on the NIH Gene Expression Omnibus under the series GSE277431.

### Assessment of virus mutations

Sequencing reads (described above) were mapped to the genome sequence of the VEEV TrD or TC-83 seed stock to assess virus mutations using CLC Genomics Workbench (Qiagen, v23). Viral SNPs were identified using Low Frequency Variant Detection with the following parameters, minimum coverage = 100, minimum count = 50, and a minimum frequency = 1.0%. Ns mutations were determined by using the Amino Acid Change tool to filter away SNPs that did not result in nonsynonymous changes.

### Construction of mutations and testing of antiviral resistance using a *trans-*replicase assay

We tested BDGR-49 resistance of wild-type VEEV TrD and NSP2 mutants to BDGR-49 using a *trans*-replicase assay based (28) on VEEV-TrD (kind gift by Dr. Andres Merits). The two plasmid systems use one plasmid to express nsp1234 (pCMV-1234) and one reporter plasmid (pHSPoll-FG) to produce full-length RNA (genomic RNA) serving as the template for firefly luciferase (Fluc) and *Gaussia* luciferase (Gluc) expression. As a negative control for replication, we used a plasmid (pCMV-1234^GAA^) in which motif C in the NSP4 (polymerase) is mutated from GDD to GAA, rendering the replication complex inactive. Mutations were engineered into NSP2/4 to create four additional plasmids: pCMV-1234_NSP4:Q210R, pCMV-1234_NSP2:Y102C, pCMV-1234_NSP2:Y102H, and pCMV-1234_NSP2:L156Q. Site-directed mutagenesis was performed using Genscript, and mutations were confirmed using Sanger sequencing. All plasmids were amplified in *E. coli* TOP10 and purified to Genscript’s Research Grade standard ensuring low endotoxin levels. For the assay, 35,000 293T cells were seeded per well in 96-well plates. After 16 h, BDGR-49, diluted 2-fold from 3 to 0.006 µM for a total of 10 concentrations (*n* = 4), was added to the seeded plate, and then, the plasmids were transfected using FuGene 6 (Promega). Expressions of Fluc and Gluc were measured 18 h post-transfection using the Dual-Luciferase Reporter Assay System (Promega) using a GloMax Navigator Microplate Luminometer (Promega).

### Mouse study design and sample collection for pathology

BALB/c mice (*n* = 14) were treated twice per day with either PBS or with 6 mg kg^−1^ of BDGR-49. BDGR-49 was formulated in 25% PEG400/10% Kolliphor RH40/65% water as previously described (22). Treatments were administered by subcutaneous injection starting at −2 h prior to infection on day 0 through day 7 for a total of 8 days. Mice were monitored for mortality and clinical signs twice a day until 14 dpi. At 4, 8, and 14 dpi, two mice per group were euthanized, and their brains were collected for pathology. The VEEV TrD group only had one time point on 4 dpi as the mice succumbed to infection prior to the later time points. Following euthanasia, the whole brain with the olfactory bulb was removed from each mouse and fixed in 20 mL of 10% formalin for a total of 48 h at 4°C. Mouse brains from VEEV TrD-infected or VEEV TrD-infected and BDGR-49 treated were collected on 4, 8, and 14 dpi (one cut in the midline of the brain, separate left and right were trimmed to embedded in coronal and sagittal sections separately), dehydrated with serial alcohol and xylene, and embedded in paraffin. Four-micrometer sections of the brain were cut using a Leica microtome.

### Immunohistochemical staining of mouse brains

For immunohistochemical (IHC) staining and imaging of sections, brain sections were deparaffinized and rehydrated in distilled H_2_O. Antigen retrieval was performed using Antigen Unmasking Solution, Citric based (H-3300 Vector Laboratories, USA), followed by washing with PBS, and endogenous peroxidases were quenched with 1% hydrogen peroxide in PBS for 5 min, rinsed with PBS three times, and blocked with 1% bovine serum albumin (BSA) in PBS and 0.3% Triton X-100. Sections were incubated with goat anti-VEE GP antibody (gift from Kurt Kamrud, AlphaVax, NC) at a 1:12,000 dilution overnight at 4°C in a moisture box, for rabbit AIF-1/Iba-1 antibody (NBP2-19019, Novus Biologicals), with dilution of 1:1,000, rabbit anti-GFAP antibody (NB300-141, Novus Biologicals) with a dilution of 1:2,000, rinsed with PBS three times and incubated with biotinylated rabbit anti-goat IgG at 1:200 (BA-5000, Vector Laboratories) or biotinylated horse anti-rabbit IgG (BA-1100, Vector Laboratories) for 2 h in room temperature. After rinsing three times with PBS, the sections were incubated with avidin-biotin complex (ABC) using the Vectastain ABC Elite Kit PK-6100 (Vector Laboratories. USA) at a 1:100 dilution and developed with 3,3ʹ-diaminobenzidine (Sigma-Aldrich, USA). Sections were counterstained with hematoxylin, dehydrated through serial alcohol and xylene, and then mounted using Permount Mounting Media (Thermo Fisher Scientific).

### Digital imaging and processing

All slides were scanned with an Olympus VS200 by scanning slides with a 40× objective. Images were captured and processed with the OlyVia v. 3.2 software.

### HALO image analysis process

For image analysis, slides were scanned, and the percentage area of brain that was labeled for VEEV GP antigen was determined by quantitative morphometry using the HALO Area Quantification v2.4.3 algorithm (Indica Labs, Albuquerque, NM). In an iterative process, a pathologist annotated the training regions, and after visually confirming the accuracy of results in additional sections, adjusted program settings until the positive signal was consistently correctly annotated. To further improve the artificial intelligence recognition accuracy, training cycles were repeated until the number of misrecognized regions was reduced to an acceptable level (i.e., until the analysis results did not change appreciably with the completion of further training).

### Three-dimensional structural modeling

The model of nsP2 and nsP4 was generated using AlphaFold2 under the ColabFold framework using default parameters (29, 30). The FASTA sequences of the VEEV nsP2 helicase (accession number YP_010806452, residues 184 to 415) and nsP4 polymerase (accession number YP_010806454, residues 1 to 606) from NCBI were input into the AlphaFold ColabFold server (3). The model with the highest pLDDT score was chosen for docking (29).

### Molecular docking

Docking was performed with AutoDock Vina (31, 32) (with exhaustiveness set to 128) to model the interaction between BDGR-49 and nsP2/nsP4. Before commencing the docking procedure, the protein and ligand were prepared individually. Hydrogens were added to both the ligand and receptor, and coordinates were saved into the PDBQT format, which is compatible with AutoDock Vina. The grid was then generated, centered on the chain interface, and the ligand was docked into the nsP2/nsP4 model coordinates to determine the best orientation with the lowest overall energy changes. PyMOL (33) and UCSF Chimera (34) were used for molecular visualization and figure preparation.

### Statistics

Survival curves were generated and assessed on GraphPad Prism version 10 using the Log-rank (Mantel-Cox) survival analysis, and *P* values were generated from the comparisons between the survival curve of each treatment group and the survival curve of the virus-only group. For the evaluation of infectious virus titers using the plaque assay, we used a two-way ANOVA multiple comparison analysis with Šidák multiple comparison correction, and *P* values were generated from comparisons between each treatment group on their respective sample days. For RNA-seq, differential gene expression analysis was assessed using DESeq2 in R based on gene count results generated using CLC Genomics Workbench version 23. Genes with an average gene count <5 were removed. Differential gene expression from each of the treatment groups was determined against the untreated-uninfected group for each of the sampled days. Genes that had a log_2_ fold change value ≤−1.5 and ≥1.5 and an FDR value ≤0.05 were noted as being significantly differentially expressed. Genes that were significantly differentially expressed as well as having a base mean count ≤10 were used to determine pathway activation by using Ingenuity Pathway Analysis of each treatment group on each sampled day.

## RESULTS

### Prophylactic treatment of BDGR-49 confers 100% survival to BALB/c mice, given a lethal challenge of VEEV TrD, but only increased the mean time to death for EEEV FL-93–939 or WEEV Fleming

To assess the level of protection of BDGR-49 against an intranasal challenge of a lethal dose of VEEV TrD, EEEV FL93-939, or WEEV Fleming in BALB/c mice, we measured morbidity and mortality for 14 days (Fig. 2). We chose a prophylactic treatment regimen of 6 mg kg^−1^ twice daily using a subcutaneous route of administration for a total of 6 days starting 2 h prior to exposure with virus or mock. In our prior studies using subcutaneously VEEV TrD-infected BALB/c mice, protection was provided using a subcutaneous route of administration of BDGR-49 at the 6 mg kg^−1^ twice daily for a total of 6 days (22). Sham-treated mice infected with VEEV, EEEV, or WEEV met clinical criteria for euthanasia by 5, 4, and 3 dpi, respectively (Fig. 2). Only BALB/c mice challenged with VEEV TrD and treated with BDGR-49 showed substantial protection at 100%. In contrast, BALB/c mice treated with BDGR-49 and intranasally infected with EEEV or WEEV showed a median survival of 5 and 4 days, respectively.

### Prophylactic treatment of BDGR-49 reduces VEEV in the brain of BALB/c mice

As 100% of the VEEV mice treated with BDGR-49 survived through 14 dpi (Fig. 2), we repeated an identical study with VEEV TrD with and without BDGR-49 treatment to enable evaluation of the virus load in whole brain (with main olfactory bulb) using four mice per group. BALB/c mice intranasally infected with VEEV TrD met the criteria for euthanasia on 5 dpi, and hence, we chose time points at 2, 4, and 5 dpi to measure virus load in the absence of treatment. In the VEEV TrD-BDGR-49-treated group, we chose time points at 2, 4, and 8 dpi. The viral titers in the brains of VEEV-infected mice were significantly reduced (*P* < 0.0001) by about 1 log PFU on 2 and 4 dpi in the BDGR-49 treatment groups compared with VEEV TrD-only groups (Fig. 2A). Three of the four mice challenged with VEEV TrD and treated with BDGR-49 had no detectable infectious virus in their brain on 8 dpi [at limit of detection (LOD) of the plaque assay] (Fig. 3A). Additionally, we conducted RNA-seq on these brain samples and mined the data set to define the relative levels of full-length viral RNA (Fig. 2B and 3). Similar to the plaque assay results of the brain, the levels of viral RNA on 2 and 4 dpi were significantly lowered (*P* < 0.0001) by the BDGR-49 treatment (Fig. 3B and 4). By 8 dpi, the levels of positive-sense, viral RNA genome transcripts in the brain in BDGR-49 treatment group were reduced but still present in the brain compared with plaque counts, which were at baseline levels.

### Identification and assessment of potential resistance mutations to BDGR-49 identified in VEEV TrD-exposed mouse brains

We evaluated each of the RNA-seq data sets for any potential resistance mutations that have been noted previously in cell culture assays that measure resistance of VEEV against BDGR-49 (22). Of the 24 samples, two of the mouse brains with the highest virus load on 4 and 8 dpi each had a resistance mutation (Fig. 3A). On 4 dpi, the change was a T to a C at nucleotide position 1953 in the genome, which resulted in a change in the amino acid Y102 to Y102H. The mutation had a depth of coverage of 476 and a frequency of 24.6%. The mutation observed at eight dpi was a T to A at position 2116 in the genome, which resulted in a change in the amino acid L156 to L156Q. The depth of coverage was 213 and the frequency of the nucleotide change was 95%.

To further explore the potential for resistance to BDGR-49, we chose a second, closely related virus, VEEV TC-83, which differs from VEEV TrD by 11 nucleotides, which results in one amino acid change in the nsP3, five amino acid differences in the E2, and one animo acid difference in E1 (35). Two nucleotide changes are in the noncoding region and two changes are silent. Using an identical treatment strategy, we assessed efficacy using a prophylactic treatment regimen of 6 mg kg^−1^, twice daily with BDGR-49 using a subcutaneous route of administration in C3H/HeN mice for a total of 6 days starting 2 h prior to intranasal exposure with VEEV TC-83 or mock. As noted by the survival data in Fig. 5A, the dosing regimen was suboptimal for this model system, and all mice in the survival group (*n* = 8) required euthanasia by 10 dpi. During the study, we took whole brains from mice (*n* = 4) at 3, 5, and 7 dpi and at the last day of survival, 10 dpi, for RNA-seq. One mouse in the 10 dpi group required euthanasia on 9 dpi. RNA-seq was performed on each of the brain samples (*n* = 16). The depth of coverage of VEEV TC-83 was determined (Fig. 5B and C), and the viral sequences in each brain sample were scanned for potential resistance mutations. Only one mouse on 9 dpi had a resistance mutation, which was the same site as observed in the VEEV TrD study. Specifically, we noted a T to a C at nucleotide position 2116 in the genome, which resulted in a change in the amino acid to L156Q. In this case, the frequency was 83.7% in a region with a coverage of 2054.

Using a *trans*-replicon assay for VEEV TrD (37), we assessed the two mutations we detected in mice treated with BDGR-49 at concentrations ranging from 5.9 to 3000 nM (Fig. 6). The two plasmid systems use one plasmid to express VEEV nsP1234 and one reporter plasmid, which produces genomic RNA encoding genes for firefly luciferase (Fluc) and *Gaussia* luciferase (Gluc). The IC_50_ resulting from these amino acid changes was 188 nM for Y102H mutation and 22 nM for L156Q mutation compared with the VEEV TrD wild-type virus, which was less than 6 nM. For comparison, we also evaluated two resistance mutations previously detected using *in vitro* assays, the NSP2:Y102C and NSP4:Q210R, which had IC_50_ values of 176 nM and >3,000 nM, respectively.

### BDGR-49 significantly reduces the proinflammatory response and cell death pathways in BALB/c mice intranasally infected with VEEV TrD

**Fig 7:**
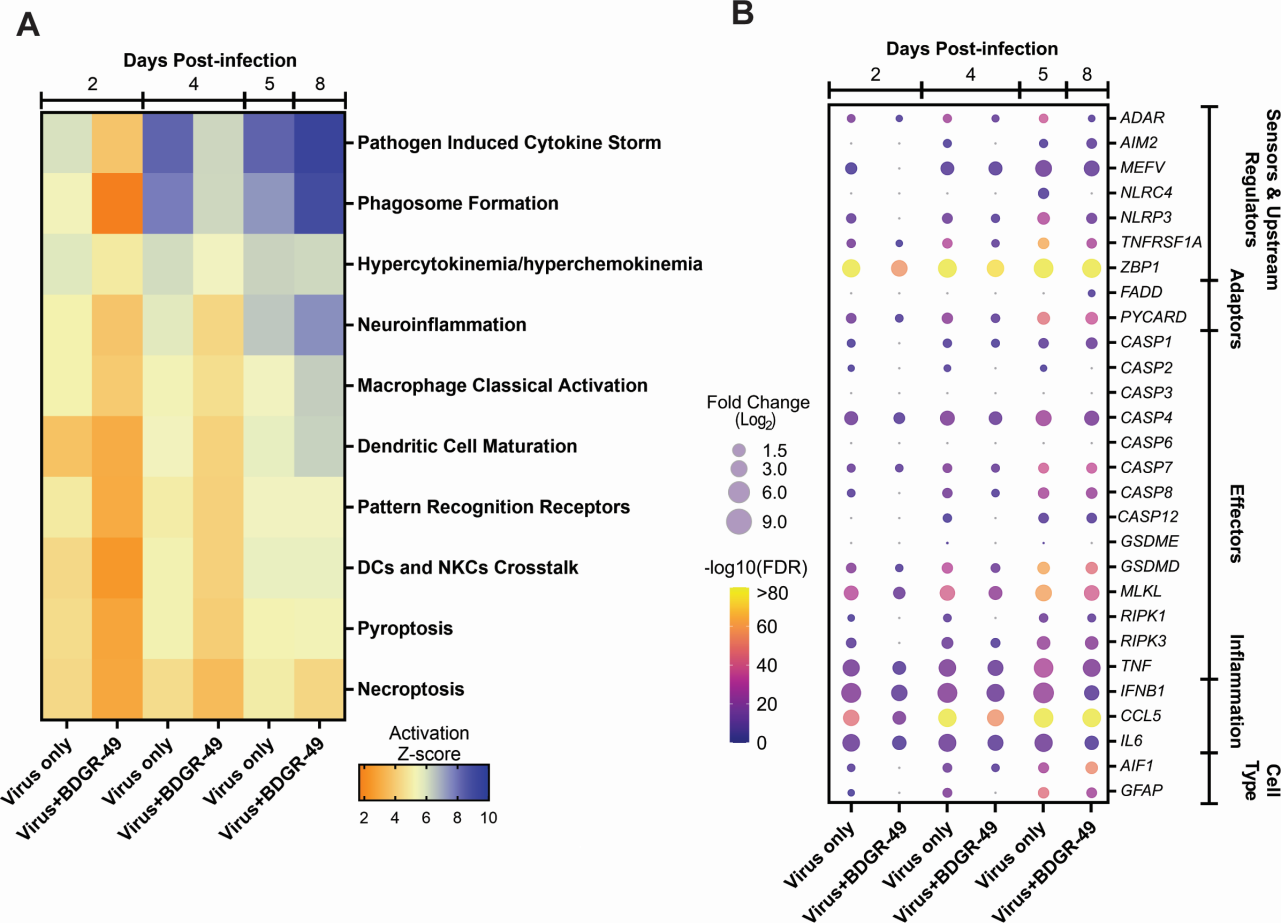
The major canonical pathways and selected genes upregulated in the brains of mice infected intranasally with VEEV-TrD and sham or BDGR-49. (**A**) An ingenuity pathway analysis based on RNA-seq data was conducted for *n* = 4 BALB/c mice from each group, VEEV-TrD treated with PBS or 6 mg kg^−1^ twice daily with BDGR-49. Color bar shows the relative activation score. (**B**) Fold change of significantly differentially expressed genes (Log_2_ fold change ≥1.5 and FDR value ≤0.05) identified in signaling pathways. RNA-seq results were assessed by comparison of VEEV TrD-infected mice, BDGR-49 or sham-treated to sham-treated, mock-infected mice. Circles show the relative log_2_ fold change and color bar shows -log_10_ FDR value.

We further mined the RNA-seq data set from the brain tissue to define the major signaling pathways of the host responses in the brains of VEEV TrD-infected, sham-treated mice compared with VEEV TrD-infected, BDGR-49-treated mice. A comparison of the most significant signaling pathways showed that the BDGR-49 treatment of VEEV TrD-infected mice resulted in a significant reduction in several canonical pathways on 2 and 4 dpi compared with the sham-treated, VEEV TrD-infected mice (Fig. 7A). Specifically, a reduction was noted in immune cell signaling pathways, such as phagosome formation, macrophage classical activation signaling pathway, and crosstalk between dendritic cells and natural killer cells. Pathways involved in cytokine signaling pathways in brains of VEEV TrD-infected, sham-treated mice increased until euthanasia on 5 dpi. VEEV TrD-infected, BDGR-49-treated mice had reduced cytokine signaling and neuroinflammation until 8 dpi when these pathways showed an upregulation when viral RNA, but not infectious virus, could be detected (Fig. 3 and Fig. 7A). Finally, signaling pathways associated with programmed cell death (PCD) pathways, pyroptosis and necroptosis, were higher VEEV TrD-infected mice-sham-treated mice on 2 and 4 dpi. Although pyroptosis signaling pathway was similar (activation score 5.2 versus 5.3) between the two groups by 5 and 8 dpi, respectively, the necroptosis signaling pathway remained higher in the VEEV TrD-infected sham-treated mice.

**Fig 8:**
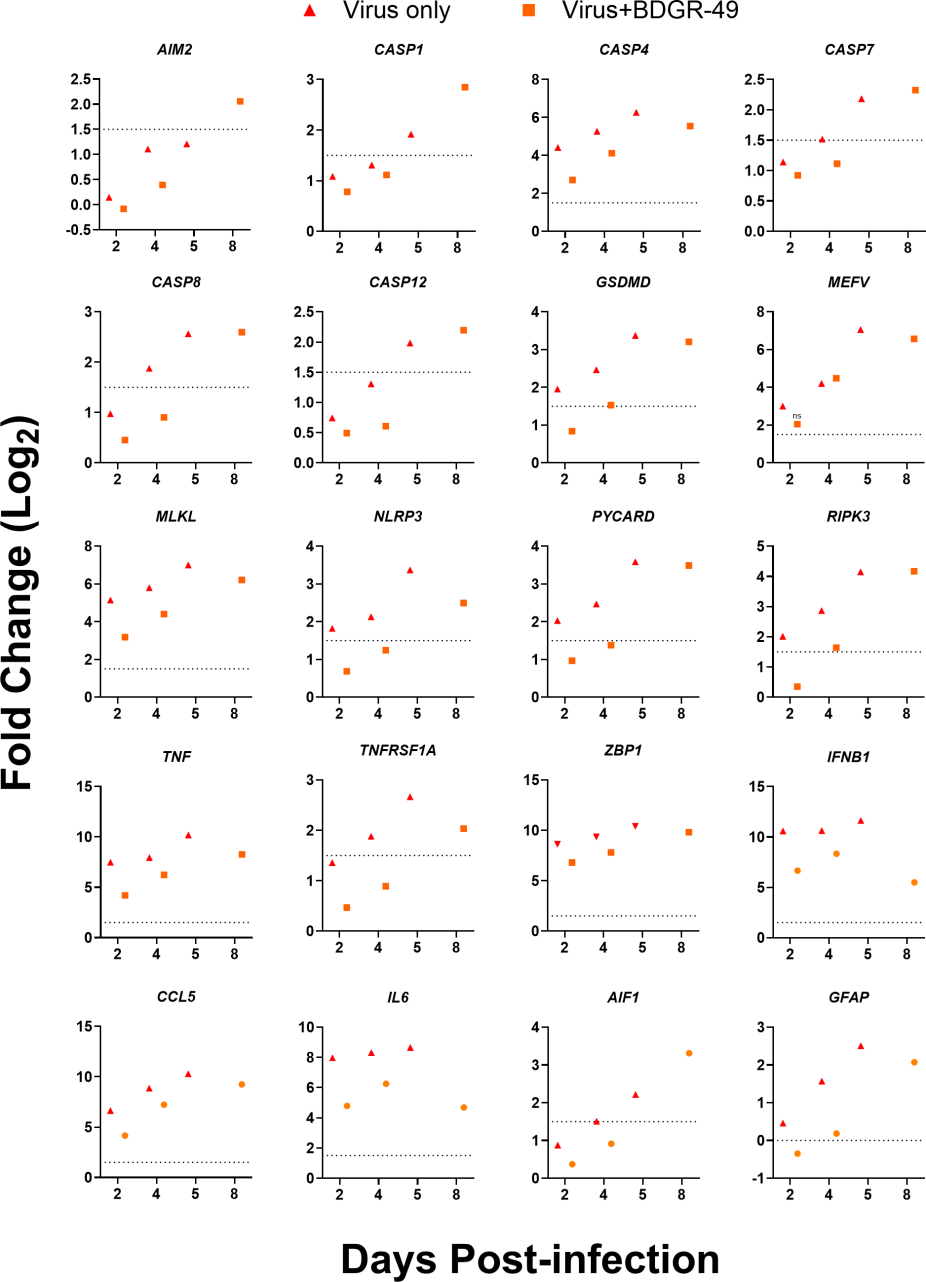
Fold change of selected significantly upregulated genes from inflammatory and cell death pathways. At 4 dpi, whole brains (including the olfactory bulb) from 5- to 6-week-old, female BALB/c mice that were infected intranasally with VEEV TrD and treated with BDGR-49 or sham (*n* = 4 per group) were harvested and processed for RNA-seq as described in material and methods. Differential gene expression was calculated using DESeq2 comparing sham or BDGR-49-treated, VEEV TrD-infected mice with mock-infected, sham-treated mice. Individual gene expression plots from genes are shown in a bubble map (Fig. 7B). The dotted line indicates a log_2_ fold change = 1.5. All gene fold change values above the dotted line have an FDR value ≤0.05 unless indicated with ns (not significant).

We noted that the inflammatory and PCD pathways, necroptosis and pyroptosis, were reduced in brains of mice infected with VEEV TrD and treated with BDGR-49 in our analyses of the RNA-seq data set (Fig. 7A). Evaluation of specific genes (Fig. 7B and Fig. 8) on 2 dpi from RNA-seq of brains from VEEV TrD-infected, sham-treated mice showed significantly elevated levels of the sensors and upstream regulators, *MEFV* (*P* = 0.018)*, NLRP3* (*P* = 1.60 × 10^−6^), and *ZBP1* (*P* = 1.82 × 10^−91^); the adaptor, *PYCARD* (*P* = 3.10 × 10^−14^); and the effectors, *CASP4* (*P* = 1.98 × 10^−12^)*, GSDMD* (*P* = 3.76 × 10^−20^)*, MLKL* (*P* = 6.04 × 10^−32^)*, RIPK3* (*P* = 1.65 × 10^−5^), and *TNF* (*P* = 1.65 × 10^−15^). On 4 dpi (Fig. 7 and 8), elevated levels of *CASP4* (*P* = 3.45 × 10^−18^), *CASP8* (*P* = 9.60 × 10^−15^), *AIF1* (*P* = 7.98 × 10^−13^), *GFAP* (*P* = 3.69 × 10^−18^), and *TNFRSF1A* (*P* = 4.24 × 10^−31^) in VEEV TrD were measured in infected, sham-treated mice. The levels of these genes on these days were reduced or not upregulated in the VEEV TrD-infected, BDGR-49-treated mice over this period, 2–4 dpi. By 8 dpi, *CASP7, CASP12, GSDMD, ZBP1, RIPK3, PYCARD,* and *CCL5* levels in VEEV TrD-infected, BDGR-49-treated mice were similar to VEEV TrD-infected, sham-treated mice on 5 dpi, except for *NLRC4*, which was upregulated only in VEEV TrD-infected, sham-treated mice (Fig. 7B). Additionally, *AIM2* was only observed in brain tissues of VEEV TrD-infected, BDGR-49-treated mice on 8 dpi (Fig. 7B and Fig. 8). The timing of expression of these genes suggests BDGR-49 treatment delayed their activation during the treatment window, and their activation began when treatment stopped after 5 dpi or 6 days of treatment. The reduction in PCD and inflammation may potentially play a role in the observed survival of VEEV TrD-infected, BDGR-49-treated mice.

### Morphometric and microscopic evaluation of pathogenesis in the brains of VEEV TrD-infected BALB/C mice with and without BDGR-49 treatment

To evaluate the extent of VEEV TrD in BDGR-49-treated and untreated mice, an experiment was designed and conducted in which two mice were sacrificed per day per group. The three groups were (i) mice challenged with PBS and sham-treated (ii), mice challenged with VEEV TrD and prophylactically treated subcutaneously with BDGR-49 twice per day at 6 mg kg^−1^, and (iii) mice challenged with VEEV TrD and sham-treated. Whole brains were taken on 4, 8, and 14 dpi and fixed in formalin. As observed previously, all mice challenged with VEEV TrD were euthanized by 5 dpi, and hence, this group does not have 8 or 14 dpi samples. Serial sections of sagittal and coronal sections of whole brains were stained with hematoxylin and eosin (H&E) (Fig. S1), probed for the presence of viral glycoprotein (GP), or probed for the ionized the calcium binding adaptor molecule 1 (Iba1).

**Fig 9:**
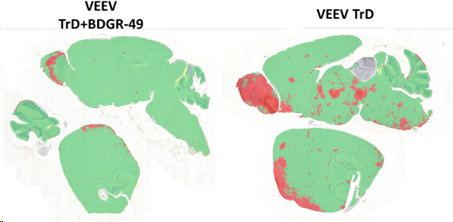
Morphometric analysis of brains from BALB/c mice intranasally infected with VEEV TrD and VEEV TrD treated with BDGR-49. Paraffin-embedded slides prepared from the brains of BALB/c mice (*n* = 2/group) were evaluated at 4 dpi for the presence of VEEV TrD glycoprotein (shown in red) by quantitative morphometry using HALO™ image analyses software as described in the Materials and Methods.

**Fig 10:**
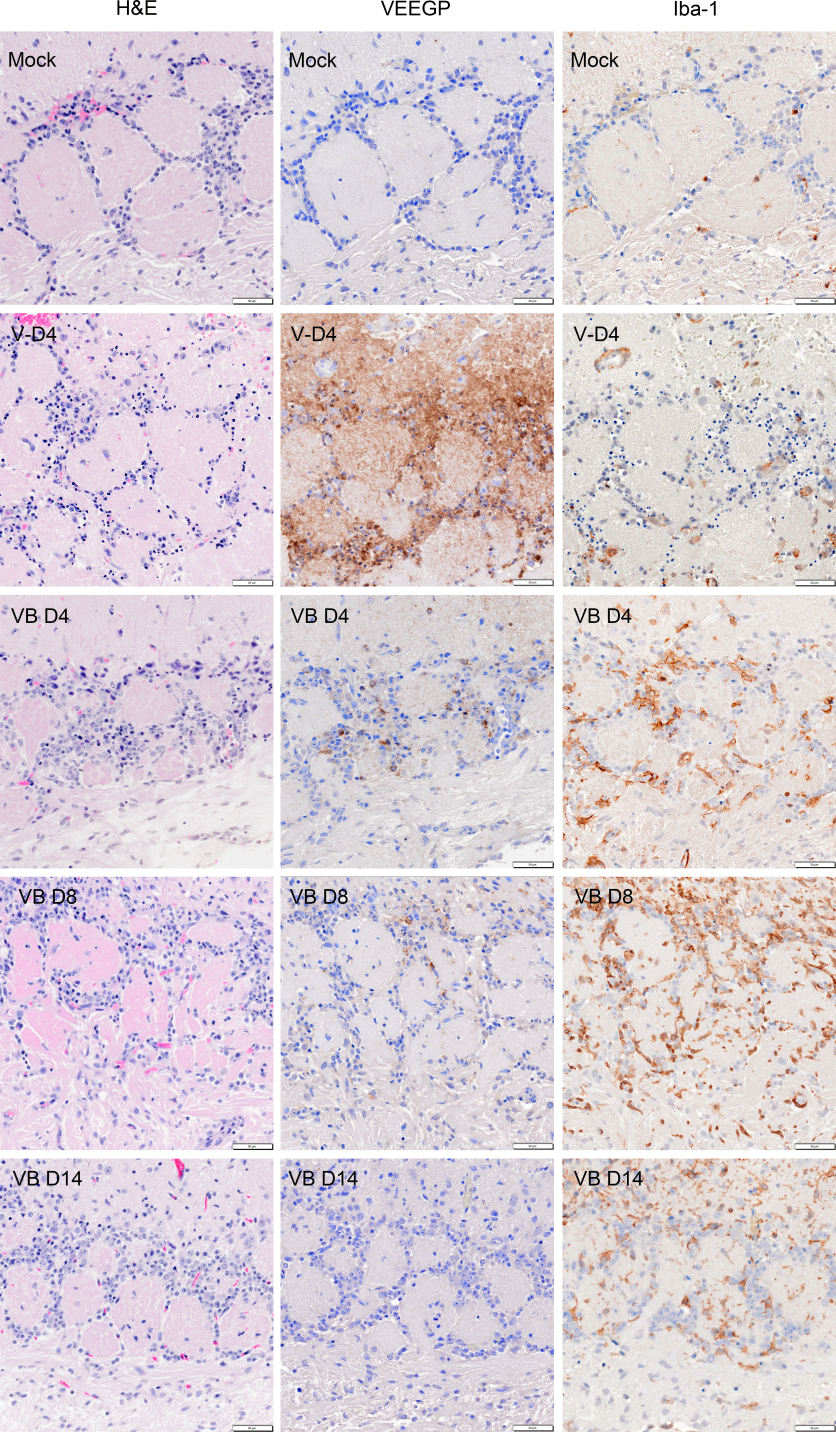
Representative H&E and immunohistochemistry images of the main olfactory bulb (MOB) from BALB/c mice that were PBS-exposed (mock-infected), untreated VEE TrD-infected (4 dpi), and VEE TrD infected treated with BDGR-49 (at 4, 8, and 14 dpi). Adjacent serial sections were stained with hematoxylin and eosin (H&E) in the first column. immunohistochemically for VEEV glycoprotein (VEE-GP) in the second column, and for a Iba-1, a marker for microglia, in the third column. In the mock-exposed samples, we observed an intact histology structure of the outer plexiform layer (opl), glomerular Layer (gl), and olfactory nerve layer (onl). In adjacent serial sections of Mock-infected tissue, no VEEV GP and scattered ramified microglia were detected. In the MOB of VEEV TrD-infected, sham-treated mice at dpi 4, we noted abundant lesions with pyknosis, karyorrhexis, mononuclear cells spread, and mild vacuolation in the glomerular layer (VD4 H&E). With VEEV GP IHC, abundant virus GP was detected (VD-4 VEEGP). Activated microglia were detected in parenchyma and around blood vessels (arrowhead, VD4 Iba-1). Mice on 4 dpi that were exposed to VEEV TrD and BDGR-49-treated showed fewer degenerating neurons and inflammatory cells than in the sham-treated, VEEV TrD-infected mice (VBD4-H&E). On 4 dpi, a reduced amount of VEEV GP (VB, VEEVGP) was detected in the neuronal soma and neuropil, and minimal neuronal destruction compared with sham-treated VEEV TrD-infected mice. The MOB of VEEV TrD infected, sham-treated mice showed numerous activated microglia (VB4-Iba-1), but VEEV TrD-infected mice treated with BDGR-49 showed markedly increased numbers of activated microglia at all 3 time points (4, 8, and 14 dpi) (VBD8-H&E). By 8 dpi, viral GP (VBD8) in VEEV TrD infected, BDGR-49 treated MOB was still detected in neurons near soma but was remarkedly reduced in neuropil (i.e., suggesting less virus in axons). At 14 dpi, no neuronal degeneration was evident, although some inflammatory cell infiltrates were present (VDB14-H&E). At 14 dpi, in these mice, only minimal VEEV GP staining was detected (VBD14-VEEGP), and microgliosis was lower than on eight dpi (VDB14-Iba-1). Scale bar = 50 μm.

**Fig 11:**
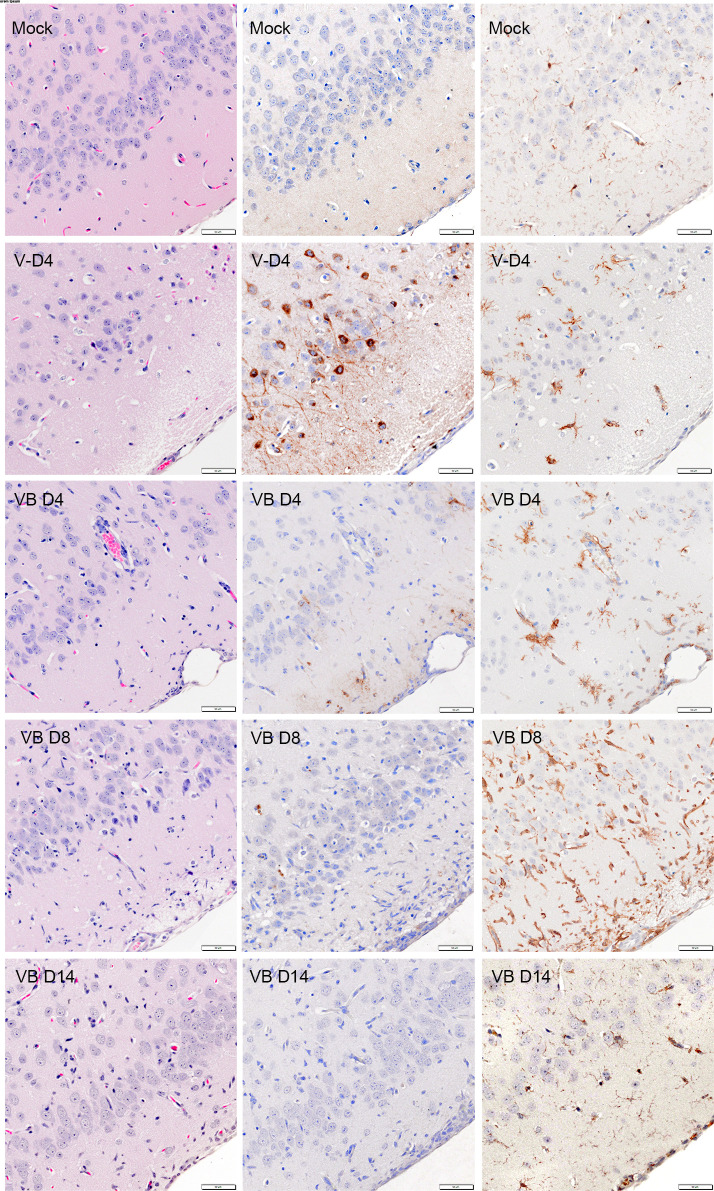
Representative H&E and immunohistochemistry images of the piriform cortex from BALB/c mice that were PBS exposed (mock-infected), untreated VEE TrD-infected (4 dpi), and VEE TrD-infected treated with BDGR-49 (at 4, 8, and 14 dpi). In the mock-infected mice, serial sections show intact neuronal, blood vessels, and meninges of piriform cortex by H&E staining (Mock-H&E). In an adjacent serial section, no viral glycoprotein (GP) antigen was detected (Mock, VEEGP). Iba-1 staining showed delicately ramified microglia (Mock-Iba-1). At four dpi in PBS-treated VEEV-TrD-infected mice, there was vacuolation of neuropil, scattered shrunken eosinophilic degenerating neurons (arrowhead) (VD4, H&E). In sequential sections, there were numerous GP-positive neurons and neuropil (VD4, VEEGP). Iba-1 IHC (DV4, Iba-1) image showed activated microglia in virus-positive areas. In contrast, VEEV-TrD-infected mice treated with BDGR-49 showed an increase in inflammatory cell infiltration in piriform cortex around blood vessels and meninges (VB4, H&E) and markedly reduced viral GP staining in neurons and neuropil (VB4, VEEGP). Activated microglia were present around blood vessels, capillaries, and within meninges in virus-positive areas (VBD4, Iba-1). By 8 dpi, H&E showed increased inflammatory cell infiltrates (VBD8, H&E) but reduced GP signal (VBD8, VEEGP), and markedly increased activated microglia (VBD8, Iba-1). At dpi 14, neuronal lesions neuronal lesions and inflammatory cell infiltrates were absent (VB14, H&E) and viral GP was not detected (VBD14, VEEGP). Iba1-positive microglia and macrophages were mostly restricted to perivascular neuropil and meninges (VBD14, Iba-1). Scale bar = 50 μm for all images.

Morphometric analysis of brains (Table 1; Fig. 9) probed for the viral GP revealed no virus-positive staining in the sham/mock (PBS) mouse group on 4, 8, or 14 dpi. VEEV TrD infection in both treated and sham-treated infected mice was most extensive in the main olfactory bulb and olfactory cortex (Fig. 9 and 10; Fig. S1). The virus spread to other areas of the brain was markedly reduced in VEEV TrD-infected, BDGR-49-treated mice. In our morphometric analyses of the brains of VEEV TrD-challenged mice (Table 1; Fig. 9), the percentage of the area positive for viral staining was 15.7%–17.4%. In the brains of VEEV TrD-challenged mice that were treated with BDGR-49, the percentage of the area positive for viral staining was 2.3%–2.4% on 4 dpi, 0%–0.9% on 8 dpi, and 0% on 14 dpi. Microscopic examination of H&E slides showed small numbers of apoptotic cells in olfactory bulb of VEEV TrD-challenged mice that were treated with BDGR-49 at 4 dpi (Fig. 10). On 4 dpi, in the untreated VEEV TrD group, numerous apoptotic cells were observed in olfactory bulb (Fig. 10) and multifocally in cortex (Fig. 11 ), midbrain, and brain stem. By 14 dpi, H&E microscopic evaluation showed extensive inflammatory cell infiltrates in the olfactory bulb, and multifocal perivascular infiltrates in other areas were noted in VEEV TrD-challenged mice that were treated with BDGR-49 (Fig. 10 and Fig. 11; Fig. S1). Extensive inflammatory activation characterized by reactive microglia in Iba-1-stained sections was observed in all mice challenged with VEEV TrD regardless of treatment (Fig. 10 and Fig. 11).

**TABLE 1:** Morphometric analysis of VEEV TrD-infected mouse brains with and without BDGR-49 treatment

Parameter	Result on day:					
	4	4	8	8	4	4
Treatment	BDGR-49	BDGR-49	BDGR-49	BDGR-49	None	None
Virus	TrD	TrD	TrD	TrD	TrD	TrD
Mouse ID number	2A107	2A108	2B109	2B110	3A113	3A113
Virus positive area (mm²)	2.22	2.25	1.37	0.87	13.90	16.91
Normal brain area (mm²)	94.87	92.49	96.28	110.70	70.32	80.44
Total assessed brain area	97.09	94.74	97.66	111.57	83.40	97.35
Glass area (mm²)	0.56508	2.2445	1.1235	1.04957	0.10168	1.418
% Virus-positive brain	2.3%	2.4%	1.4%	0.8%	15.7%	17.4%

### Molecular docking results support that BDGR-49 binds at the interface between nsP2 and nsP4

**Fig 12:**
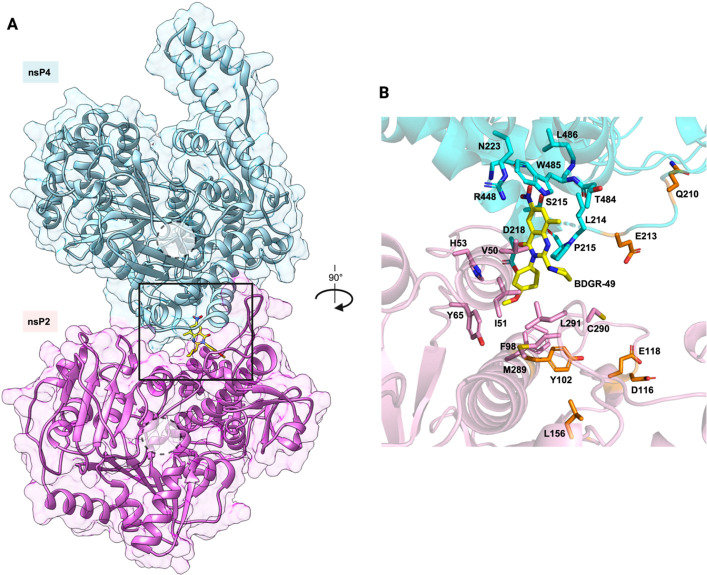
Interaction of BDGR-49 at the interface of VEEV nsP2 and nsP4. (**A**) Overview of the nsP2-nsP4 complex with bound BDGR-49 (in sticks, with C atoms in yellow), the dashed circles represent the site clefts of nsP2 and nsP4, respectively. (**B**) Close-up view of BDGR-49 and its interacting residues. Hydrogen bonds are shown as yellow dashed lines, whereas residues selected for BDGR-49 resistance are highlighted in orange.

The modeling results identified the presence of a cleft at the interface of the nsP2/nsP4 macromolecular complex, distal to the nsP4 active site, suggesting it as a potential binding pocket. The docking studies support the idea that BDGR-49 binds at the junction of nsP2 and nsP4 as shown in Fig. 12A and B shows the main interactions between BDGR-49, nsP2, and nsP4.

The residues corresponding to the identified resistance mutations—E213, Q210 in nsP4 and Y102, D116, E118, and L156 in nsP2—are not in the immediate vicinity of BDGR-49’s binding site at a distance of ~10 Å and facing the opposite side (Fig. 12B). It is thus plausible that the mutations may alter the binding site by enabling conformational changes that affect the interface architecture. Overall, the docking results suggest that the inhibition of BDGR-49 on viral synthesis may be through allosteric effects on the nsP4 and/or nsP2 activities.

We modeled the nsP2-nsP4 complexes of EEEV and WEEV using AlphaFold3. Pairwise Cα structural alignment of nsP2 and nsP4 from EEEV and WEEV with VEEV revealed that although the overall RMSD values were observed to be 0.83 Å and 1.0 Å, respectively, significant structural rearrangements were noted in the loop regions. Structurally, the EEEV and WEEV complexes exhibit greater similarity to each other compared with the VEEV one (Fig. S2). Notably, major conformational changes were identified in the nsP4 loop regions near the BDGR-49 binding site. Sequence alignment (Fig. S3) indicates that A190 in VEEV nsP4 is replaced by a proline in EEEV and WEEV nsP4, introducing a kink in the α-helix. This prompts a different orientation of the subsequent alpha helix and loop, which results in a positioning of L214 in EEEV and WEEV that would clash with the docking pose of BDGR-49 in the VEEV nsP2-nsP4 complex. Thus, binding of BDGR-49 in nsP2-nsP4 complexes of EEEV and WEEV could be compromised by these changes. However, potency *in vitro* is not a limitation as reported using Vero 76 antiviral assays where BDGR-49 reduces infectious titer by 5.8 logs by EEEV compared with >7 logs (limit of detection) reduction for VEEV (22).

## DISCUSSION

There is general agreement in the literature that the pathology of VEEV in the CNS is similar in animal models and humans (8, 17, 38), and hence, we focused on the infection of the brain of mice in our evaluation of BDGR-49. BALB/c mice intranasally exposed to 10× LD_50_ of VEEV, EEEV, or WEEV reached criteria for humane euthanasia at 5, 4, or 3 dpi, respectively. The median survival (5 dpi) of the mice intranasally infected with VEEV TrD was similar to our prior studies using the inguinal route of exposure (22). In our prior studies, prophylactic treatment of BALB/c mice with BDGR-49 that were subcutaneously VEEV TrD infected resulted in 75% protection when mice were treated twice per day for 6 days with 12 mg kg^−1^ or 6 mg kg^−1^. Surprisingly, we achieved slightly better protection, 100% versus the 75% in the inguinal infection model, in mice intranasally infected with VEEV TrD and treated prophylactically with BDGR-49, twice-daily using the subcutaneous route, for 6 days with 6 mg kg^−1^. Our prior studies of subcutaneous exposure to EEEV and treated twice-daily prophylactic subcutaneous treatment with BDGR-49 for 6 days, conferred 25% protection. In contrast, no mice survived intranasal exposure to EEEV when using the same treatment regimen. In the subcutaneous infection of mice with VEEV, the virus infects dendritic and macrophage cells. Dendritic cells traffic to the draining lymph nodes where VEEV replicates in lymphocytes, and within 12 h, the virus may transit into the bloodstream where it can access and infect brain, lung, spleen, and other organs (39). Given the greater systemic nature of subcutaneous exposure of VEEV compared with the intranasal, the studies herein and our prior studies (22) suggest that the pharmacokinetic exposures of BDGR-49 should be higher in subcutaneous versus intranasal VEEV exposure to achieve 100% protection. In contrast, for EEEV, a comparison of BDGR-49 efficacy studies in mice exposed intranasally (in this paper) or previously by the subcutaneous route (22) suggests the opposite in that BDGR-49 had a greater protective effect against subcutaneous exposures (25%) versus intranasal (0%). This may be due to more rapid and efficient entry of EEEV into the CNS via the olfactory tract following intranasal infection. Moreover, unlike VEEV, EEEV does not infect dendritic cells or macrophages, and therefore, its ability to replicate within lymphoid tissues is minimal (39). Future pharmacodynamic studies will be required to ascertain the optimal approach for treatments of both routes of exposure based on these notable differences in disease etiology.

A major thrust of our efficacy studies has sought to measure the effect of BDGR-49 on the reduction of viral load in the brain. Infectious virus and viral RNA were reduced 1–2 logs in the brains of VEEV-TrD intranasally infected, BDGR-49 treated BALB/c mice, which was similar to the levels observed in studies with VEEV TrD inguinal infected, BDGR-49-treated mice (22). By 8 dpi, no virus was detected in 75% of the BDGR-49-treated BALB/c mice at the 6 mg kg^−1^ dose. The levels of infectious virus in the brain following intranasal exposure (10^7.5^ PFU/mL) are higher than previously observed for inguinal exposure (10^4^–10^6^ PFU/mL). Although we have previously shown that 48 mg kg^−1^ day^−1^ BDGR-49 will reduce viral load to the limit of detection on 4 dpi, we used a lower dose of 12 mg kg^−1^ day^−1^ to provide a comparison of our prior efforts at a similar treatment regimen (22) and allow for a treatment dose that would allow detection of the VEEV TrD RNA transcripts so that we could determine whether any resistance of VEEV TrD to BDGR-49 might emerge as previously discovered in *in vitro* dose escalation resistance assays using VEEV TC-83 (22). In these *in vitro* resistance assays with VEEV TC-83, resistance was mapped using next-generation sequencing in nsp2 at Y102C, D116N, and E118V and nsp4 at Y87F, Q210R, and Q201K (22). In mice treated with BDGR-49, we detected two mutations in the VEEV TrD NSP2, Y102H in one mouse at 4 dpi and a L156Q in another mouse at 8 dpi, but none of the other more common nonsynonymous mutations were previously observed (22, 26). The amount of viral RNA detected by RNA-seq was extremely low for the L156Q with only a depth of coverage of 213. We reconstructed the L156Q mutant into a *trans*-replicase assay (28) using a two plasmid system, VEEV TrD NSP1234 and vRNA reporter, and measured an EC_50_ = 22 nM compared with <<6 nM for the wild-type VEEV TrD in the presence of increasing concentrations of BDGR-49. We expect that this level of resistance would be easily overcome with a higher dose of BDGR-49 but that would need to be empirically determined. In AlphaFold models congruent with the published high-resolution structure of the core RNA replicase of Chikungunya virus (CHIKV) (40), the L156Q mutation lies in the 1B region near the stalk domain, mutation Y102H, and upstream of the RecA1 and RecA2 helicase domains (Fig. 12). At present, little is known about the 1B region and its function. It is suggested to play a role in stabilizing the different conformational arrangements of nsP2 during interactions with RNA (41). The proximity of these two amino acids is similar in the structure of CHIKV nsP2 (40). The L156 is a conserved amino acid across multiple alphaviruses including VEEV, EEEV, WEEV, Ross River virus, and CHIKV. Within the nsP2:nsP4 structure, the Y102, L156, and Q210 are in close proximity (Fig. 12).

The key inflammatory markers noted in this current RNA-seq study for the VEEV TrD-infected, sham-treated BALB/c mice were similar to those we reported previously in C3H/HeN mice intranasally infected by VEEV TC-83 (16) and also those noted by others (42–45). We show that BDGR-49 treatment of VEEV TrD-infected BALB/c mice resulted in lowered gene expression levels in the brain of several key inflammatory markers, *IFNB1*, *TNF*, *IL6*, and *CCL5*. This agrees with the finding that the upregulation of *TNF-α* and *CCL5* in plasma correlates with pathology during subcutaneous infection of BALB/c mice with VEEV TrD (43). Together, these data suggest that the pro-inflammatory cytokines CCL5, TNF-α, IL-6, IL-1β, and/or IFN may serve as biomarkers within the serum to monitor and thereby assess the progress of antiviral treatment in future preclinical studies in mice. Although there may be unique cytokines in sera for specific strains of VEEV in combination with different mouse strains, our work and others suggest that identification of a common set across VEEV strains is possible, and our future studies will continue to define this possibility, given the importance of biomarkers in sera to evaluate efficacy in preclinical and clinical studies.

Histopathological examination of the brain showed that neurons are clearly undergoing cell death. Cell death may be driven by several mechanisms such as programmed cell death (PCD) or nonprogrammed cell death (non-PCD). Programmed cell death can be divided into lytic and non-lytic cell death with panoptosis, necroptosis, and pyroptosis pathways mediating lytic mechanisms and apoptosis mediating the nonlytic mechanism. Non-programmed cell death (Non-PCD) refers to necrosis where irreversible cell damage and final cell death are caused by physical or chemical stimulation. NK cells play a role in VEEV pathogenesis as demonstrated by their depletion which improves survival of lethal VEEV infection in mice (21). Apoptosis has also been proposed to play a role, although these studies were conducted in cell culture with VEEV (46). At present, we have no insight into the role that PCD or Non-PCD mechanisms may play in cell death of neurons noted within the brains of the VEEV-mouse models; however, in our prior study of VEEV TC-83 infection of C3H/HeN mice, we noted a significant upregulation of genes associated with PCD pathways. In examination of the PCD pathways for untreated, VEEV TrD-infected BALB/c mice, we noted the upregulation genes associated with pyroptosis and necroptosis. Specifically, *GSDMD* and *IL-1B* were both upregulated, which suggests the activation of pyroptosis in the brain (47). GSDMD functions by forming pores in cells releasing inflammatory proteins (48–51). The lack of *CASP3* upregulation in our studies suggests that pyroptosis occurred through a *CASP3*-independent manner as caspase-3 inhibits GSDMD activity (52). *ZBP1* was upregulated in VEEV TrD-infected mice. ZBP1 is triggered by viral RNA as reported for influenza A virus and SARS-CoV-2 (53, 54), and this protein can also activate the inflammasome, which can activate caspase-1 and 4 and induce pyroptosis. *CASP1* and *4* were both upregulated. Additionally, we noted high expression levels of *MLKL* and *RIPK3*, which are associated with necroptosis, but a lack of *RIPK1*, which may suggest that necroptosis occurs through a RIPK1-independent manner (55, 56). Necroptosis may also be activated by ZBP1 (57). Although much work remains to dissect the main driver of cell death in the PCD pathways, it is clear that ZBP1 plays a role (57). The lack of proapoptotic genes such as *BAK1* and *BAX* (58) also suggests that apoptosis was not activated in our RNA-seq studies. In VEEV TrD-infected, BDGR-49-treated mice, the levels of the genes *NLRP3*, *ZBP1, PYCARD*, *GSDMD, MLKL, RIPK3*, *CASP4*, and *CASP8*, associated with PCD, were lower. Further studies will be needed to confirm the contributions of these pathways to neuronal cell death and CNS pathogenesis.

In conclusion, efficacy studies of BDGR-49 at a constant dose level of 6 mg kg^−1^ given subcutaneously twice daily in BALB/c mice exposed to intranasal exposure of VEEV, EEEV, or WEEV provide 100% protection only to VEEV-infected mice. Higher dose regimens for treatment of mice, intranasally challenged with EEEV and WEEV will be explored in future studies. Evaluation of viral load, inflammation, cell death, and pathology of VEEV-infected mice that were treated with BDGR-49 suggested substantial improvement of each of these endpoints. As BDGR-49 does not provide broad spectrum antiviral efficacy, our future studies look to improvement of this scaffold through further structure-activity relationship studies. Of importance to the development of a broad spectrum antiviral for the treatment of VEEV, EEEV, and WEEV is the recognition that the disease etiology resulting from the route of exposure, mouse species, and viral strain will all need to be considered in testing and evaluation. Moreover, gaps remain in our understanding of pathogenesis both during infection and recovery to optimize treatment regimens.

## Data Availability

All RNASeq data from the studies herein are publicly available under the Gene Expression Omnibus series GSE255795, GSE277300, and GSE277431.

## SUPPLEMENTAL MATERIAL

Supplemental figures jvi.01679-24-s0001.docx Figures S1 to S3.

ASM does not own the copyrights to Supplemental Material that may be linked to, or accessed through, an article. The authors have granted ASM a non-exclusive, world-wide license to publish the Supplemental Material files. Please contact the corresponding author directly for reuse.
