# Supplementary material for: The antiviral BDGR-49 provides protection from lethal, neurotropic Venezuelan equine encephalitis virus intranasal infection in mice
Source: J Virol. 2025 Feb 12;99(3):e01679-24. doi: 10.1128/jvi.01679-24 (PMC11916738; doi:10.1128/jvi.01679-24)
Supplement: Supplemental figures — Figures S1 to S3. [file jvi.01679-24-s0001.docx]

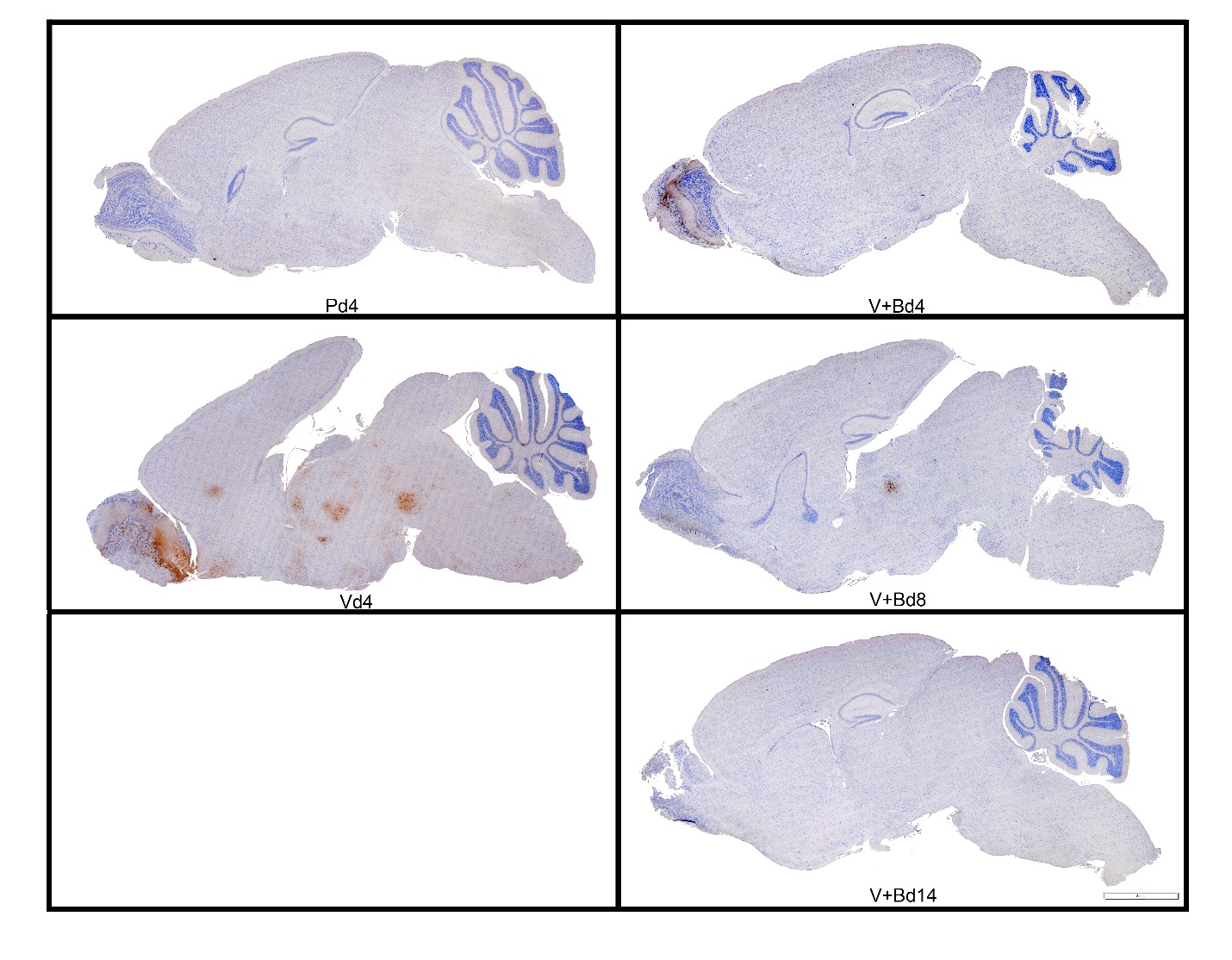
**Supplemental Figure 1.** **Representative sagittal brain sections probed for viral glycoprotein antigen.** Sagittal sections of brain samples from each group were stained simultaneously for viral glycoprotein (GP). At 4 dpi, the mock infected brain showed no GP staining (Pd4). Brains from VEEV TrD infected mice infected at 4 dpi shows GP spread throughout the major areas. The most intense GP signals were detected within the main olfactory bulb, and piriform cortex with GP staining also noted in the olfactory nucleus, cerebral cortex, corpus callosum, striatum, hippocampus, thalamus, hypothalamus, superior colliculus, midbrain, pons, medulla, cervical spinal cord and cerebellum (Vd4). In the brains of mice infected with VEEV TrD and treated with BDGR 49, GP was mostly detected in the olfactory bulb, but the number of infected neurons were tremendously decreased compared with the sham-treated brain (Pd4). Scattered infected neurons were also detected at the olfactory nuclear, piriform cortex, thalamus and spinal cord (V+Bd4). At 8 dpi in the brain of VEEV TrD infected, BDGR-49 treated mice, limited VEEV GP were detected in olfactory bulb, piriform cortex and one focal area in the thalamus nuclear (V+Bd8). At 14 dpi, no viral antigen was detected in neurons of the VEEV TrD infected, BDGR-49 treated mice (V+BD14). Scalebars=2mm.


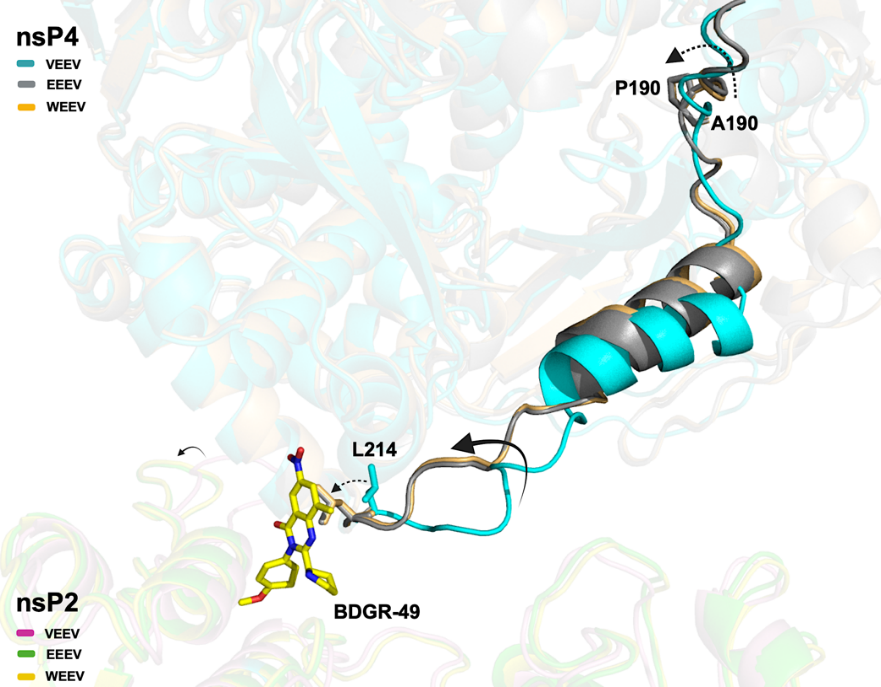


**Supplemental Figure 2.** Pairwise structural alignment of nsP2 and nsP4 of EEEV and WEEV with VEEV revealed overall RMSD values of 0.83Å and 1.0 Å respectively, significant structural rearrangements observed within the loop regions.

**Supplemental Figure 3- Multiple sequence alignment of nsP4 from VEEV with WEEV and EEEV.** Figure prepared using ENDscript 3.
